# A randomised controlled trial of extended immersion in multi-method continuing simulation to prepare senior medical students for practice as junior doctors

**DOI:** 10.1186/1472-6920-14-90

**Published:** 2014-05-02

**Authors:** Gary D Rogers, Harry W McConnell, Nicole Jones de Rooy, Fiona Ellem, Marise Lombard

**Affiliations:** 1 School of Medicine, Griffith University, Gold Coast, Queensland 4222, Australia; 2 Health Institute for the Development of Education and Scholarship, Griffith University, Gold Coast, Queensland 4222, Australia; 3 School of Pharmacy, Griffith University, Gold Coast, Queensland 4222, Australia

## Abstract

**Background:**

Many commencing junior doctors worldwide feel ill-prepared to deal with their new responsibilities, particularly prescribing. Simulation has been widely utilised in medical education, but the use of extended multi-method simulation to emulate the junior doctor experience has rarely been reported.

**Methods:**

A randomised controlled trial compared students who underwent two, week-long, extended simulations, several months apart (Intervention), with students who attended related workshops and seminars alone (Control), for a range of outcome measures.

**Results:**

Eighty-four third year students in a graduate-entry medical program were randomised, and 82 completed the study. At the end of the first week, Intervention students scored a mean of 75% on a prescribing test, compared with 70% for Control students (P = 0.02) and Intervention teams initiated cardiac compressions a mean of 29.1 seconds into a resuscitation test scenario, compared with 70.1 seconds for Control teams (P < 0.01). At the beginning of the second week, an average of nine months later, a significant difference was maintained in relation to the prescribing test only (78% vs 70%, P < 0.01).

At the end of the second week, significant Intervention vs Control differences were seen on knowledge and reasoning tests, a further prescribing test (71% vs 63% [P < 0.01]) and a paediatric resuscitation scenario test (252 seconds to initiation of fluid resuscitation vs 339 seconds [P = 0.05]).

**Conclusions:**

The study demonstrated long-term retention of improved prescribing skills, and an immediate effect on knowledge acquisition, reasoning and resuscitation skills, from contextualising learning activities through extended multi-method simulation.

## Background

Tell me and I will forget,

Show me and I may remember,

Involve me and I will understand.

Xun Zi (c312–230 BCE).

Around the world, accreditation bodies charge medical schools with preparing students so that on graduation they are ‘competent to practise safely and effectively as interns’ [1], or the equivalent role in their local healthcare system [2,3]. However, the transition from medical student to junior doctor is a significant one and there is evidence from multiple countries that many commencing interns feel ill-prepared to deal with their new responsibilities [4-11]. Areas of particular concern in relation to intern preparedness appear to vary somewhat between jurisdictions, but include: clinical decision making [4,6,8], the management of emergencies [4,6,7,9,11], communication of difficult news [6,7,10], and the performance of practical procedures [4,7,9].

One of the key qualitative differences between the roles of medical students and interns in the clinical environment is that, although their practice remains supervised, interns are empowered to prescribe drugs without the necessity for physical countersigning by a senior colleague. Hilmer’s group reported that Australian graduates from multiple medical schools appear, both subjectively and objectively, to be poorly equipped for this task, giving rise to real concern about patient safety [12]. These findings were echoed in several of the recent broader studies of intern preparedness from other settings [4,9,10,13], as well as earlier specific prescribing studies by Pearson and colleagues [14,15].

One jurisdiction, New Zealand, has approached this issue through the establishment of a transitional ‘trainee intern’ year. This is undertaken in the sixth year of undergraduate-entry medical programs at the country’s two medical schools, following ‘barrier’ assessment. ‘Trainee interns’ receive a tax free educational grant that approximates a salary and emphasises the transitional nature of the role between education and employment [16]. Dare and colleagues showed that students who have completed this year report having gained experience in all of the identified areas of concern described above and rate their own competence in many of them significantly more highly than do students who are yet to commence it [17]. This approach clearly has promise for improving intern preparedness, but implementing it in larger health systems, where four-year, graduate-entry medical degrees are the norm, poses very real practical difficulties.

The most important challenge for medical educators in preparing students to take on the responsibilities of junior doctors is the tension between providing the experience of making patient care decisions (and observing their consequences) on the one hand and ensuring the safety of patients on the other [18]. As Gordon and colleagues have pointed out, simulation methodologies allow students to ‘“practice” medicine without risk’ to real patients [19].

In a comprehensive review of the evidence supporting the use of simulation in medical education, Okuda and colleagues referenced more than 100 papers demonstrating educational benefit from simulation methodologies [20]. All of the studies cited, however, assessed learning in relation to a particular set of skills or the management of a particular condition and utilised short, isolated clinical scenarios or sometimes disembodied part-task trainers. An earlier review by Issenberg’s group identified features of simulation activities that best facilitate learning, including: providing feedback, curriculum integration, capturing clinical variation and providing a controlled environment [21].

Ziv and colleagues have reviewed the use of simulation from the patient safety perspective and suggest that there is an ethical imperative for medical educators to utilise this approach [22], while Cook and Triola have explored the potential for computer-based ‘virtual patients’ to enhance clinical reasoning [23].

The Australian Medical Association contends, with some justification, that ‘the most productive clinical placements occur when medical students are included as members of the hospital/clinical team, are assigned an appropriate level of responsibility for patients and tasks, and are actively included in the team’s educational and review activities’ [24]. Such involvement has been seen as being essential in developing students’ clinical reasoning abilities, as well as preparing them to manage their own patients, with lower levels of supervision, after graduation.

As far as we can determine, however, only one report to date has examined the use of *extended* live-action simulation of realistic junior doctor work, which might provide learning opportunities that approximate the experience of extended participation in a clinical team and taking ongoing personal responsibility for the care of patients [25]. This study, undertaken by Laack and colleagues, reported only students’ self-perceived preparedness for internship, however, and did not measure learning outcomes.

In this paper we report on a randomised trial of the educational effectiveness of a program comprising two periods of extended immersion in a multi-method, realistic continuing simulation of junior hospital doctor life, provided to senior students at an Australian medical school.

### CLEIMS

The Griffith University Medical Program is a graduate-entry, four year course. In Year 3 and Year 4 of the Program, students are placed in clinical settings for all of their time, except for one ‘in-school week’ each year. In these weeks, a proportion of the cohort (approximately 25 students at a time in Year 3 and 50 students in Year 4) returns to the medical school campus for an intensive week that is intended to focus on the skills, knowledge and understanding that are important to the junior doctor role but difficult for students to acquire safely in real clinical settings.

Originally, these in-school weeks comprised unconnected seminars and workshops but student engagement was poor and the Clinical Learning through Extended Immersion in Medical Simulation (CLEIMS) methodology was developed in an attempt to emphasise the relevance of the weeks to future practice.

In CLEIMS, learners experience an accurate, continuing simulation of a realistic clinical story from their likely future professional lives, interspersed with more traditional seminars and workshops on topics raised by the simulation. We postulated that their experience of managing the simulated patients (SPs) would make learners aware of the current gaps in their relevant knowledge, understanding or skills and would thus provide strong, immediate motivation for them to attend to and engage with the related ‘just in time’ workshop or seminar. These include intensive interactive tutorials focusing on prescribing and medication safety, based on those described by Coombes and colleagues [26], as well as traditional mannequin-based resuscitation training and practical workshops on topics such as fluid resuscitation, fundoscopy and airway management. After each learning activity the students return to the simulation and continue the patients’ management utilising their newly-acquired skills and understanding.

Prior to commencing their in-school week, students are randomly allocated into simulated clinical teams of four or five. In order to emulate medical team structure, without compromising the requirement for student groups to make their own decisions, one member of each group is randomly chosen to be the ‘registrar’ (leader of the junior doctor team in the Australian system) while the others play the role of ‘interns’. In Year 3, each team is required to assess and manage its own live trained SP. Investigation results ‘become available’ at a realistic interval after students have ‘ordered’ the test and teams are required to complete documentation such as medication charts, fluid orders and case notes as they would in a real clinical setting.

The story proceeds through the week, with the patient being played on some occasions by the SP, while at others her progress is followed ‘virtually’, with the group’s facilitator providing updated information at intervals. Most of the story unfolds ‘real time’ but ‘time lapses’ are also used to enable an extended clinical course to be covered in the single week available. On several occasions in the week, a second SP plays the part of the patient’s relative.

At one point the patient becomes acutely and unexpectedly unwell. Each team undertakes the management of this event in succession with the patient represented on this occasion by a high fidelity simulation mannequin.

Each member of the team is required to go ‘on call’ overnight for one night of the week. On some of these nights, the student ‘on call’ receives an automated text message at unsociable hours, whereupon they are required to go online and manage developments in the patient’s condition through interactive internet modules. On other evenings, members of the academic team make live telephone calls to the students ‘on call’, in the role of the registered nurse responsible for the patient’s care. The following morning students report back the events of the night to their teams and justify the clinical decisions they have made.

In Year 4, each student team manages a total of eight SPs with interconnecting stories, in ‘real time’, over the course of the week. We achieve this by rotating teams through ‘stations’ where they encounter, manage and assess a particular SP for 30-60 minutes. As student teams rotate to the next patient, the SP ‘resets’ their story so that the next team encounters them in the same situation.

In both years of the program, pharmacy students join the scenario at appropriate times for realistic simulations of the interaction between junior doctors and their pharmacist colleagues.

Throughout each week, we require students to keep a textual journal, through which they have the opportunity to process and reflect on their experiences in the simulation. In addition, at several points, the SPs ‘break character’ to provide feedback to students about their human skills. We video record each team’s management of the medical emergencies and undertake traditional ‘debriefing’ reflection and feedback later whilst reviewing the video recording [27]. Finally, at the end of the week, we hold a wrap up session where students can discuss the key decision points in the patient’s story to clarify and optimise their learning.

The extended immersive simulation methodology was piloted with Year 3 medical students in 2009. Initial evaluations indicated that participating students rated the approach as highly effective in helping them to learn (mean rating of 6.4 on a 7-point Likert scale) but, since the program is quite resource-intensive, we felt that more substantial evidence of educational effectiveness would be required to ensure its institutional sustainability. Thus we designed and implemented a randomised controlled education trial.

The research question for the study was:

Does the contextualising effect of immersion in extended continuing simulation improve acquisition or retention of skills, knowledge and understanding from the associated seminars and workshops?

## Methods

The protocol was reviewed and approved by the Griffith University Human Research Ethics Committee.

**Figure 1:**
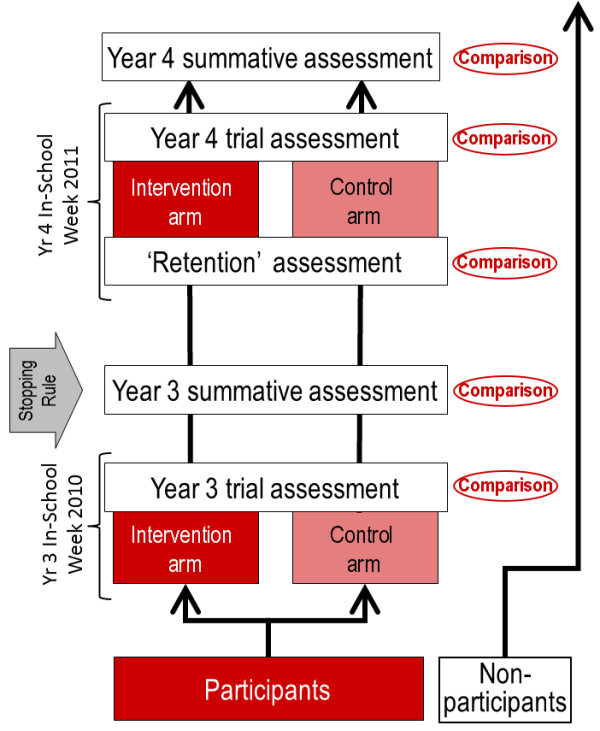
**Study design.** See separate file: RogersEtAlFigure1.png.

We approached students entering Year 3 of the Griffith University Medical Program in 2010 through a presentation at their orientation session and invited them to participate in the study. We provided full details of the randomised nature of the study and its two-year duration at this briefing. Figure 1 summarises the design of the study.

One sixth of students in the Year 3 cohort undertake the in-school week on each of six occasions throughout the academic year. In the week prior to each scheduled in-school week, we contacted students who had elected to participate in the study by email to determine whether they still wished to take part. Once this had been confirmed, participating students rostered for the particular week were randomised 1:1 by coin toss to either an Intervention or Control group. Participants from each group were then randomly allocated into teams of 3 – 5 (depending on the number of consenting participants in that rotation).

Teams in the Intervention group undertook the full CLEIMS methodology as described above. Teams in the Control group (and study non-participants) participated in the same seminars and workshops as Intervention students, but did not undertake the associated extended simulation. We instructed participants in the Intervention arm to keep the content of the simulation confidential and specifically not discuss it with Control participants.

On the Friday of the in-school week, all participants undertook a range of additional non-summative assessments. We chose outcome measures to assess key aspects of educational effectiveness in relation to preparation for practice (see Table 1).

**Table 1:** Outcome measures

**Point in study**	**Outcome measure name**	**Details**	**Outcomes compared**
End of in-school week in Year 3	MCQ3	20x ‘choose one from five’ Multiple Choice Questions based on the content of the seminars and workshops undertaken during the Year 3 in-school week by participants in both study groups.	Number correct out of 20, for each individual participant.
	SCQ3	50x Script Concordance Questions, formulated and scored using the method described by Fournier’s group [28], to assess clinical reasoning in relation to conditions covered in the seminars and workshops undertaken during the Year 3 in-school week by participants in both study groups.	Total score out of 50, for each individual participant.
	PS3	A structured prescribing exercise comprising a printed scenario describing the circumstances of a typical hospital medical admission (a 64 year old woman with an infective exacerbation of COPD), including a GP referral letter and ‘registrar directions’, with the following request to the participant: ‘Please fill out a standard hospital drug chart to prescribe her usual medications and follow your registrar’s directions. You may add any other therapy you think is clinically appropriate at this time.’ Scored according to a structured score matrix by an expert (blinded to participants’ study group) in relation to therapeutic decisions, medication safety and technical aspects of chart completion. All of these issues were covered in workshops undertaken during the Year 3 in-school week by participants in both study groups.	Total score out of 100, for each individual participant.
	RS3	Team resuscitation exercise in which each participant team is distracted by being led to believe that they are going to be tested on urinary catheterisation, then a ‘nurse’ in the adjoining room bursts in seeking their help, saying that her patient has ‘stopped breathing’. The team is then required to assess the patient (a simulation mannequin) and initiate cardiopulmonary resuscitation. Whole exercise is video recorded and timed from the recording *post hoc*.	Time from call for help to initiation of cardiac compressions, for each team.
End of Year 3 academic year	SDP3	Final summative assessment scores for the *Doctor and the Patient* (clinical skills) theme for Year 3 of the Medical Program (comprised of Mini-CEX assessments from each clinical rotation, multiple choice and ‘mini-case’ short answer questions).	Final score out of 269 marks for each individual participant.
	SOV3	Final summative assessment scores for Year 3 of the entire Medical Program (comprised of SDP3 plus multiple choice, ‘mini-case’ short answer questions and assignments in the other themes).	Final score out of 672 marks, for each individual participant.
**Beginning** of in-school week in Year 4 (‘retention’ measures from previous year)	MCQR	Identical to MCQ3 but administered an average of nine months later to measure ‘retention’ of knowledge and understanding acquired in Year 3 in-school week.	Number correct out of 20, for each individual participant.
	SCQR	Identical to SCQ3 but administered an average of nine months later to measure ‘retention’ of knowledge and understanding acquired in Year 3 in-school week.	Total score out of 50, for each individual participant.
	PSR	Identical to PS3 but administered an average of nine months later to measure ‘retention’ of knowledge, understanding and skills acquired in Year 3 in-school week.	Total score out of 100, for each individual participant.
	RSR	Identical to exercise to RS3 but undertaken in Year 4 allocation teams, an average of nine months later to measure ‘retention’ of knowledge, understanding and skills acquired in Year 3 in-school week and its transferability to new team settings.	Time from call for help to initiation of cardiac compressions, for each Year 4 team.
**End** of in-school week in Year 4	MCQ4	25x ‘choose one from five’ Multiple Choice Questions based on the content of the seminars and workshops undertaken during the Year 4 in-school week by participants in both study groups.	Number correct out of 25, for each individual participant.
	SCQ4	30x Script Concordance Questions, formulated and scored using the method described by Fournier’s group [28], to assess clinical reasoning in relation to conditions covered in the seminars and workshops undertaken during the Year 4 in-school week by participants in both study groups.	Total score out of 30, for each individual participant.
	PS4	A similar exercise to PS3 but based on a more complex patient scenario involving anticoagulant usage, cardiac and respiratory disease. Scored by same method as PS3.	Total score out of 100, for each individual participant.
	RS4	Team resuscitation exercise in which each participant team plays the part of an emergency department team receiving a seriously ill child who has collapsed following prolonged vomiting and diarrhoea at home and has been brought in by ambulance. The team is required to receive hand over from the ‘paramedic’, assess the patient (a simulation mannequin) who was pulseless and apnoeic, then initiate appropriate urgent treatment. Whole exercise is video recorded and timed from the recording post hoc.	Time from entering room to initiation of fluid resuscitation, for each team.
Summative assessment in Year 4 (after conclusion of all in-school weeks)	SDP4	Final summative assessment scores for the *Doctor and the Patient* (clinical skills) theme for Year 4 of the Medical Program (comprised of Mini-CEX assessments from each clinical rotation and multiple stations in an Objective Structured Clinical Examination [OSCE]).	Final score out of 204.5 marks for each individual participant.
	SOS4	Total score on all stations in the final OSCE for the Medical Program (including stations from all three themes using the OSCE as an assessment tool).	Final score out of 211.5 marks for each individual participant.
	SOV4	Final summative assessment scores for Year 4 of the entire Medical Program (comprised of SDP4 plus multiple choice, ‘mini-case’ short answer questions, OSCE stations and assignments in the other themes).	Final score out of 516.5 marks for each individual participant.

At the end of 2010, we also compared students in the two groups in relation to their summative assessment in the medical program. We had written a pre-determined ‘stopping rule’ (analogous to the role of a data safety and management board in a randomised clinical trial) into the protocol in order to avoid needless educational disadvantage to Control group students had the intervention proven unexpectedly to be very highly effective. We found no significant differences between the two participant groups in relation to any aspect of their summative assessment for Year 3 of the Medical Program and on this basis we continued the trial into the second year.

In 2011, individual participants remained in their assigned study groups (Intervention or Control) on the basis of the original randomisation but we assigned them (randomly) to new teams in advance of their rostered in-school week (since members of each original team were not necessary undertaking the same rotation as their former colleagues in the second year). As in the first year of the study, Intervention participants undertook the complete CLEIMS program, while Control group members (and study non-participants) attended the associated workshops and seminars, but not the extended simulation that sought to contextualise them.

At the *beginning* of each in-school week in 2011, all participants undertook the identical assessments to those they had completed at the end of their 2010 week, in order to assess their ‘retention’ of skills, knowledge or understanding acquired in the previous year. While the scenario undertaken for the practical team activity was also the same, participants undertook this assessment with members of their ‘new’ team rather than the team in which they had been assessed previously, as described above.

At the *end* of each in-school week in 2011, participants in both the Intervention and Control groups undertook further, new, study assessments based on the content of the Year 4 program, as described in Table 1.

Participants completed reflective journals in relation to their learning during their participation in the in-school weeks in both years of the study and we will present qualitative analysis of these journals in a separate publication.

We also compared participants’ results in final summative assessment for the Medical Program (undertaken in the middle of Year 4, after all students had completed the in-school week) in relation to the two study groups.

We undertook the study opportunistically in the real-world setting of medical student education. Although the study was planned and outcome measures were determined prospectively, no formal sample size calculations were undertaken.

Mean scores for each outcome measure were compared using t-tests (with Welch’s correction where the standard deviations of the two groups were significantly different) utilising the GraphPad ‘Instat’ statistical program (GraphPad Software, Inc., La Jolla, CA) with a probability of 0.05 being used to identify significant differences.

## Results

139 students commenced Year 3 of the Medical Program in 2010 and were invited to participate. Of these, 95 initially consented to take part. Eleven students withdrew from the study when re-contacted prior to their Year 3 in-school week (and thus prior to randomisation) leaving 84 participants who commenced the study and were randomised.

Forty-five participants were randomised to the Intervention group and 39 to the Control group.

Table 2 confirms that the randomisation was effective, since the two study groups did not differ significantly from each other in relation to any measured parameter prior to commencement of the study.

**Table 2:** Effectiveness of randomisation: comparison of baseline characteristics of intervention and control arm participants

**Characteristic**	**Intervention (n = 45)**	**Control (n = 39)**	**P value for difference**
Gender: number (%) male	19 (42%)	20 (51%)	NS*
Age at beginning of study: mean (in years)	26.7	26.4	NS**
Higher degree on entry^†^: number (%)	6 (13%)	6 (15%)	NS*
Prior healthcare worker^††^: number (%)	10 (22%)	5 (13%)	NS*
GAMSAT^‡^ score on entry: mean	61.1	60.1	NS**
GPA^‡‡^ on entry: mean	6.53	6.33	NS**
Ever failed a year^¶^: number (%)	1 (2%)	2 (5%)	NS*
Year 1 overall summative score: mean (out of 1000)	732	724	NS**
Year 1 clinical skills summative score: mean (out of 250)	185	182	NS**
Year 2 overall summative score: mean (out of 1000)	692	690	NS**
Year 2 clinical skills summative score: mean (out of 250)	159	160	NS**

Notes: * = by Fisher’s Exact Test; ** = by *t*-test, † = students who entered the graduate medical program with a prior honours, masters or doctoral degree; †† = students who had practiced in another health profession (pharmacy, physiotherapy or nursing) prior to entry into medicine; ‡ = Graduate Australian Medical School Admissions Test overall score, ‡‡ = Grade Point Average for student’s previous degree on a 7 point scale, ¶ = students who had failed any year in the medical program prior to the study.

No further participants withdrew after randomisation but one Control group participant failed to progress from Year 3 to Year 4 and one Intervention group participant took a leave of absence from the Medical Program in 2011, meaning that no further data were collected in relation to these two after the Year 3 summative assessment.

The results of comparison between participants in the two groups at each assessment point in the trial are summarised in Table 3.

**Table 3:** Between-group comparisons for each outcome measure

**Outcome measure name (see Table **1**)**	**Intervention group**			**Control group**			**Maximum possible score**	**Difference**^**† **^**in mean score (%) or time**	**P value**
	**Mean score or time (%)**	**SD score or time**	**n**	**Mean score or time (%)**	**SD score or time**	**n**			
End of Year 3 week									
MCQ3	13.1 (66%)	2.0	44	12.6 (63%)	1.9	36	20	0.5 (3%)	NS
SCQ3	29.5 (59%)	3.7	44	29.0 (58%)	3.7	36	50	0.5 (1%)	NS
PS3	75.4 (75%)	9.3	44	70.1 (70%)	11.2	36	100	5.3 (5%)	0.02
RS3	29.1 seconds	11.4 seconds	14	70.1 seconds	28.4 seconds	12	-	41 seconds	<0.01*
Year 3 summative assessment									
SDP3	202.2 (75%)	10.3	45	199.6 (74%)	12.0	39	269	2.6 (1%)	NS
SOV3	484.7 (72%)	26.8	45	480.5 (72%)	24.5	39	672	4.2 (1%)	NS
Immediately before Year 4 week (‘retention’ analysis)									
MCQR	11.4 (57%)	2.0	43	10.8 (54%)	2.2	38	20	0.6 (3%)	NS
SCQR	30.4 (61%)	3.1	43	30.0 (60%)	3.4	38	50	0.4 (1%)	NS
PSR	77.9 (78%)	6.3	43	70.4 (70%)	10.7	38	100	7.5 (8%)	<0.01*
RSR	35.8 seconds	12.7 seconds	10	46.0 seconds	14.1 seconds	10	-	10.2 seconds	NS
End of Year 4 week									
MCQ4	15.0 (60%)	2.5	43	13.3 (53%)	2.5	35	25	1.7 (7%)	<0.01
SCQ4	18.5 (62%)	2.1	43	17.3 (58%)	2.5	35	30	1.2 (4%)	0.02
PS4	70.8 (71%)	7.1	43	62.7 (63%)	10.0	35	100	8.1 (8%)	<0.01*
RS4	252.0 seconds	113.9 seconds	10	339.2 seconds	64.5 seconds	10	-	87.1 seconds	0.05
Year 4 summative assessment									
SDP4	124.7 (61%)	16.5	44	127.1 (62%)	9.2	38	204.5	-2.4 (-1%)	NS*
SOS4	115.2 (54%)	15.7	44	116.8 (55%)	9.9	38	211.5	-1.6 (-1%)	NS*
SOV4	342.0 (66%)	24.4	44	340.8 (66%)	18.6	38	516.5	1.2 (0%)	NS

Notes: n = number of individuals or (for RS scores) number of teams (small numbers of participants failed to attend for some study assessments leading to individual ‘n’ values lower than the number of participants in the study at the time); *SD* = standard deviation; all P values calculated with (independent group) t-tests except those marked *, where t-tests with Welch’s correction were used due to significantly different standard deviations between study groups; † = positive value for difference indicates Intervention group superior, negative value indicates Control group superior; scores and times rounded to one decimal place, P values rounded to two decimal places, percentages rounded to nearest whole percent.

Participation in the extended simulation program was associated with a significantly higher mean score in the prescribing exercise among individual participants at the end of the Year 3 in-school week. Teams of Intervention group participants also initiated first cardiac compressions a statistically and clinically significant 41 seconds earlier, on average, than teams of Control group students, despite both groups having undergone the same formal workshop in cardiopulmonary resuscitation the previous day. No significant difference in the acquisition of knowledge, understanding or clinical reasoning between the groups, as measured by multiple choice and script concordance tests, was evident at this stage of the trial, however.

Prior to commencement of the Year 4 in-school week, we found no significant difference between the two groups in relation to retention of knowledge or reasoning, as measured by repeat administration of the same multiple choice and script concordance tests. The significant difference in performance on the prescribing assessment between the two groups remained however, indicating retention of improved prescribing skills over an average period of nine months. At this point, teams of Intervention group participants initiated cardiac compressions an average of only 10 seconds earlier in the resuscitation exercise than their Control group counterparts and this was not a statistically significant difference.

At the conclusion of the second in-school week in Year 4, participants in the Intervention arm demonstrated improved acquisition of new knowledge and understanding, as evidenced by significantly higher mean scores on both multiple choice and script concordance questions related to material covered in the seminars and workshops that both groups had attended during the week. At this point in the trial, Intervention group participants again achieved significantly higher scores on a new prescribing exercise at an individual level and, working in teams, initiated fluid resuscitation a clinically and statistically significant 87 seconds earlier, on average, than their Control group counterparts in a simulation with an infant in cardiovascular collapse secondary to extreme dehydration.

No significant difference in summative assessment scores was seen between the groups in either year of the trial however.

## Discussion

We report on a randomised controlled trial undertaken to determine the educational impact of extended immersion in multi-method continuing clinical simulation undertaken in order to prepare medical students for their role as junior doctors. The study utilised a randomised methodology where the Control group received conventional, but still practical and interactive, seminars and workshops on key tasks and topics of relevance to the intern role. For the Intervention group these educational activities were contextualised through the use of a realistic extended simulation of their future professional lives.

Drawing on the experiential pedagogical tradition that began with John Dewey in the 1930s and was further developed by Carl Rogers, Malcolm Knowles and David Kolb in the succeeding decades, CLEIMS aims to contextualise students’ learning through a technique that clinicians would recognise as a supported form of ‘deep end therapy’. The program takes as its theoretical underpinning the work of Burns and Gentry [29], inspired by Lowenstein’s conception of the ‘curiosity gap’ [30]. This approach posits that a ‘tension to learn’ is created when students can appreciate a ‘manageable gap’ between their current state of knowledge or skill and the state that they aspire to achieve. Burns and Gentry argue that ‘a very powerful intrinsic motivator is a revealed awareness of a deficiency in a quality that is valued as a part of one’s self worth’ [29]. Medical students are clearly deeply invested in becoming competent junior doctors and extended simulated patient care experiences, where the consequences of their skill and knowledge gaps can be demonstrated safely, might be expected to generate a powerful ‘tension to learn’.

The extended simulation methodology was associated with a modest but significant and persistent positive impact on students’ prescribing skills that was additional to the benefit directly consequent on participation in the associated prescribing workshops already demonstrated in a (non-randomised) controlled trial by Coombes and colleagues [26]. The efficacy of prescribing interventions (particularly the WHO Good Prescribing Guide) has been established in a number of randomised controlled trials with ‘no-intervention’ control groups [31]. Tonkin and colleagues went further to establish the benefit of scenario-based interactive pedagogies for this purpose, compared with didactic intervention sessions covering the same material [32]. Our study deepens understanding of this area, by demonstrating that contextualisation of interactive prescribing workshops through embedding them in an extended simulation enhances their efficacy still further.

Three qualitative literature reviews underline the effectiveness of simulation as a methodology for health care workers to learn resuscitation skills but highlight the heterogeneity of study design, which renders quantitative data aggregation from multiple studies impossible [33-35]. No previous studies have been identified that examine the impact of contextualising simulation-based resuscitation training within an extended clinical simulation. Our findings support the general impression provided by the literature, in that they show that immersive simulation-based resuscitation training is markedly superior to lower fidelity mannequin- and workshop-based learning alone in relation to the acquisition of resuscitation skills. Further work will be required determine whether embedding this learning experience within an extended clinical simulation offers additional benefit compared with isolated scenario-based training, as our study design could not identify such a difference in relation to the resuscitation skills outcome. No definite effect on the retention of resuscitation skills was associated the extended simulation in our study.

We found that while a first week-long simulation involving the care of single patient did not impact significantly on the acquisition of related knowledge or clinical reasoning skills among Year 3 medical students, a subsequent second week, involving multiple simulated patients, in the fourth year of medical studies, was associated with small but significant improvements in both of these outcomes. This appears to be the first time that a difference in knowledge or clinical reasoning ability, as opposed to observable skills, has been demonstrated to be associated with participation in a simulation methodology among medical students through a randomised controlled trial. Seybert and colleagues have demonstrated that the use of high fidelity mannequin simulations were associated with knowledge acquisition in relation to cardiovascular pharmacotherapeutics among pharmacy students, but their study had an uncontrolled pre-test/post-test design [36]. Levett-Jones and collaborators recently compared the impact of medium-fidelity with high-fidelity mannequin training on the performance of nursing students on a multiple choice test related to the management of a hospital patient with fluid overload [37]. Their study did not identify any significant improvement in knowledge in either arm of their study and, as at the first time point in our trial, there was no difference in acquisition or retention of knowledge between the two experimental arms. The demonstration of improved acquisition of knowledge and clinical reasoning skills associated with the second week of simulation, several months after the first, in our study suggests that there may be a magnifying effect related to repeated extended simulation training or alternatively that the more complex and challenging simulations involving multiple patients in the Year 4 CLEIMS program generated a greater ‘tension to learn’ than the simpler Year 3 experience [29].

Despite the educational impacts seen in a number of the output measures, no significant difference was seen in final summative assessment scores between the study arms in either year of the trial. This outcome is not surprising, given that the program comprises only one week in each full year of clinical learning that is being assessed and the effect size would need to be very large indeed to be evident in final summative assessment. The persistent positive effect on prescribing skills seen at the ‘retention’ trial assessment suggests that participation in the program may well be associated with a meaningful benefit in this area, but such an effect would have been unlikely to be identified through current medical school summative assessment techniques. Further studies should focus on identifying whether the effects of the approach translate into improved patient outcomes in subsequent practice.

The study has provided high level evidence for the educational effectiveness of an extended multi-method program for medical students that simulates future practice as a junior doctor. Its beneficial effect on prescribing skills persists for a least several months.

The validity of the study’s findings may be limited by the fact that double blinding was impossible since it was clear to participants into which arm they had been randomised. We did use single blinding (of assessors) for the assessment of participants’ performance in the prescribing exercises, however. Since numerical scoring of the multiple choice and script concordance questions, as well as timing of the resuscitation outcomes from video recordings, was highly objective, markers for these items were not blinded, but this omission may raise some question about validity.

The CLEIMS methodology incorporates the key features that Issenberg and colleagues identified as critical to facilitating student learning through simulation, namely providing feedback, curriculum integration, capturing clinical variation and providing a controlled environment [21]. To this it adds the element of *extended* immersive continuing scenarios that enable learners to make their own clinical decisions, experience their outcomes and manage care over the course of an illness, without risk to real patients.

The design of the study emulated that of a ‘phase III’ clinical trial in that the addition of the ‘intervention’ was compared with an ‘optimised background’ or ‘standard of care’ control condition. This design confirms efficacy of the intervention but does not compare its effectiveness with other possible additional ‘treatments’ and it is possible that it is not the use of extended simulation *per se* but the additional time spent that brought about the differences observed. Since simulated patient management was the only educational activity undertaken in the extra time, however, it seems likely that the study question can be answered in the affirmative.

## Conclusions

This study has shown that extended immersive simulation has educational impact and may provide an important supplement to experiential learning in real clinical settings to prepare medical students for the junior doctor role. On this basis it has been included on a permanent basis in the Griffith University Medical Program.

The approach’s impact on the quality of student prescribing has proven to be persistent, at least for several months. Whether this likely benefit to patient safety justifies the considerable cost of the program, especially in terms of academic and facilitator time, will need to be modelled in a formal cost-benefit analysis currently underway.

Other potential benefits, such as impacts on student confidence and affective learning identified through qualitative analysis of student journals will be reported in a separate publication.

## Competing interests

All of the authors are employed (GDR, HWM, FE & ML on a paid basis and NJdeR as an unpaid Research Fellow) by Griffith University, which will meet the publication fee and the reputation of which may be enhanced by publication of this article. Otherwise, the authors have no competing interests.

## Authors’ contributions

GDR, HWM, NJdeR and FE conceived and wrote the initial version of the educational program that is the subject of the study, in collaboration with the development team acknowledged below. GDR conceived the study and all authors participated in its design. All authors contributed to the conduct of the study including data gathering. GDR performed the statistical analysis with assistance as acknowledged below. GDR, HWM and NJdeR drafted the manuscript, which was then critiqued by the other authors. All authors read and approved the final manuscript.

## Pre-publication history

The pre-publication history for this paper can be accessed here:

http://www.biomedcentral.com/1472-6920/14/90/prepub
